# Associations of Total and Domain-Specific Sedentary Time With Type 2 Diabetes in Taiwanese Older Adults

**DOI:** 10.2188/jea.JE20150095

**Published:** 2016-07-05

**Authors:** Ming-Chun Hsueh, Yung Liao, Shao-Hsi Chang

**Affiliations:** 1 Department of Physical Education, National Taiwan Normal University, Taipei, Taiwan; 2 Department of Health Promotion and Health Education, National Taiwan Normal University, Taipei, Taiwan

**Keywords:** sedentary lifestyle, domain-specific, TV viewing, type 2 diabetes, older adults

## Abstract

**Background:**

The increasing prevalence of type 2 diabetes in older adults has become a public health concern. We investigated the associations of total and domain-specific sedentary time with risk of type 2 diabetes in older adults.

**Methods:**

The sample comprised 1046 older people (aged ≥65 years). Analyses were performed using cross-sectional data collected via computer-assisted telephone-based interviews in 2014. Data on six self-reported domains of sedentary time (Measure of Older Adults’ Sedentary Time), type 2 diabetes status, and sociodemographic variables were included in the study. Binary logistic regression analysis was performed to calculate the adjusted odds ratios (ORs) and 95% confidence intervals (CIs) for total and individual sedentary behavior components and likelihood of type 2 diabetes.

**Results:**

A total of 17.5% of the participants reported type 2 diabetes. No significant associations were found between total sitting time and risk of type 2 diabetes, after controlling for confounding factors. After total sedentary behavior was stratified into six domains, only watching television for more than 2 hours per day was associated with higher odds of type 2 diabetes (OR 1.56; 95% CI, 1.10–2.21), but no significant associations were found between other domains of sedentary behavior (computer use, reading, socializing, transport, and hobbies) and risk of type 2 diabetes.

**Conclusions:**

These findings suggest that, among domain-specific sedentary behavior, excessive television viewing might increase the risk of type 2 diabetes among older adults more than other forms of sedentary behavior.

## INTRODUCTION

Type 2 diabetes has escalated to epidemic proportions worldwide,^1^ and its prevention has become a global public health priority.^2^^,^^3^ Type 2 diabetes is caused by the ineffective use of insulin by the body^4^ and has been linked to reduced life expectancy and increased risk of mortality.^5^ According to the World Health Organization (WHO), the prevalence of type 2 diabetes in Asian regions (10%–15%) is higher than in European and American regions (8% and 9%, respectively).^4^ The prevalence of type 2 diabetes has increased in the Taiwanese population, particularly in older adults, and was estimated to be 20.5% in 2013.^6^ To develop effective type 2 diabetes prevention initiatives, more clearly understanding the modifiable behavioral risk factors of type 2 diabetes in older adults is critical.

Sedentary behavior is defined as any waking behavior characterized by low energy expenditure (≤1.5 metabolic equivalent tasks [METs]) while in a sitting or reclining posture.^7^ Most previous studies examining sedentary behavior and type 2 diabetes have examined a specific domain of sedentary behavior, television viewing, and found a positive relationship between time spent viewing television and risk of type 2 diabetes in adults.^8^^–^^12^ Several prospective studies have reported that greater baseline television viewing time was associated with an increased incidence rate of type 2 diabetes in adults in the United States^8^^,^^9^ and Germany.^10^ Moreover, a meta-analysis concluded that people who viewed an additional 2 hours of television per day were 1.20 times more likely to have type 2 diabetes, and a linear dose-response relationship was also observed.^11^ To our knowledge, only one study examined other domains of sedentary behavior; the research reported that more occupational and transport-related sedentary time were significantly associated with a higher risk of type 2 diabetes in adults in the United States.^12^

However, previous studies examining associations between sedentary behavior and type 2 diabetes are limited in several crucial aspects. First, most studies have investigated adult populations, but few studies have focused on older adults (≥65 years), which is particularly noteworthy, as sedentary time has been found to increase with age.^13^ Second, although the significance of examining domain-specific sedentary behavior with health outcome has been emphasized for developing more effective interventions,^14^^,^^15^ most previous studies have assessed only television viewing, with little research examining the relationships between type 2 diabetes and other domains of sedentary behavior (such as time spent using computers, reading, socializing, or on transportation). Third, to our knowledge, no studies have examined the associations of both total and domain-specific sedentary time and the risk of type 2 diabetes. Further examining total sedentary time in Taiwan is particularly crucial because the Taiwanese population has a higher prevalence of total sitting time (over 6 h per day), according to an international prevalence study on sitting.^16^ Thus, preliminary investigation of total and domain-specific sedentary are critical for preventing type 2 diabetes. Fourth, most existing studies are from the United States^9^^,^^12^ and Germany,^10^ with a limited amount of data reported from Asia countries, particularly Taiwan, which has a high prevalence of type 2 diabetes among people aged 65 years or older. Because of these limitations of past research, the present study examined data from older adults in Taiwan to investigate the associations of total and domain-specific sedentary time with type 2 diabetes.

## METHODS

### Participants and data collection

A survey of people aged 65 years or older was conducted using a computer-assisted telephone interview system in two regions (Taipei City and Chiayi County) of Taiwan in July and August 2014. Sampling was performed through a random-digit-dialing telephone-based survey of households with landline telephones. All calls were conducted by an experienced telephone research service company. The potential target population comprised 468 922 older adults. Taipei City was estimated to have an elder population of 380 527 and an area of 271.8 km^2^, and Chiayi County was estimated to have an elder population of 88 395 and an area of 1903.6 km^2^.

Older adults were selected by performing stratified random sampling. The required sample size for this study was calculated using a 95% confidence level and 3% confidence interval (CI) to be 1068 older adults. Experienced interviewers administered a standardized questionnaire. All interviewers practiced administering telephone population surveys and received 2 days of training before the start of each survey. A total of 1714 older adults were telephoned, 1095 of whom completed the survey (response rate: 63.9%); after data cleaning, 1046 responses were deemed valid for analysis. The maximum time of each interview was not longer than 30 minutes for the validity. Only interviews that were completed within 30 minutes were included in the analysis. No incentive was offered to respondents. Verbal informed consent was obtained before the start of the interviews, and the study protocol was approved by the Ethics Committee of National Taiwan University (201309ES003).

### Measures

#### Outcome variable

The outcome variable was the status of type 2 diabetes (yes or no) as determined by an affirmative response to a question, which has been use Taiwanese Chronic Disease Survey of the Health Promotion Administration, Ministry of Health and Welfare.^6^ Participants were asked: “Has a doctor, nurse, or other health professional ever told you that you have type 2 diabetes, or do you use anti-diabetic medications?” The validity of this item has been verified in a subsample of this study population.^9^^,^^10^^,^^12^

#### Exposure variable

Sedentary behavior was assessed using the Measure of Older Adults’ Sedentary Time (MOST) questionnaire^17^ published by the Sedentary Behavior Research Network (http://www.sedentarybehaviour.org/sedentary-behaviour-questionnaires/) for use in assessing time spent sitting during common behaviors by older adults. The MOST questionnaire was administered using a 7-item 1-week recall to provide domain-specific sedentary time. Questions asked participants to report on activities they performed during the last week while they were sitting or lying down (not including time spent in bed) and to report the total time spent performing each activity. The 7 individual sedentary items were (a) watching television or a video or DVD, (b) using a computer, (c) reading, (d) socializing with friends or family, (e) traveling using a motor vehicle or public transport, (f) hobbies, and (g) any other activities performed while sitting or lying down. The amount of time throughout the week spent performing each activity was converted into minutes per day, and the sum of total sedentary time was calculated as minutes per day. The original summary measure was shown to have acceptable test-retest reliability (intraclass correlation coefficient [ICC] 0.52; 95% CI, 0.27–0.70; 48 participants with mean age of 73 years). Among the items, the test-retest reliability was excellent for television viewing (Spearman ρ = 0.78; 95% CI, 0.63–0.89), computer use (ρ = 0.90; 95% CI, 0.83–0.94), and reading (ρ = 0.77; 95% CI, 0.62–0.86); acceptable for hobbies (ρ = 0.61; 95% CI, 0.39–0.76); poor for socializing (ρ < 0.45; 95% CI, 0.11–0.60) and transport (ρ = 0.45; 95% CI, 0.19–0.65); relatively low for other sedentary behavior (ρ = 0.23; 95% CI, 0.38–0.74); and modestly valid (ρ = 0.30; 95% CI, 0.02–0.54) evaluated against an accelerometer-assessed counts-per-day instrument.^17^

For this study, the MOST questionnaire was translated into Chinese in accordance with the WHO process of instrument translation and adaptation.^18^ A pretest was administered to 53 older adults who were not part of the main study. The pretest respondents were asked if they understood all of the words and if they found any words or expressions unacceptable or offensive. A retest of the questionnaire was conducted 1 week later. Total sedentary time exhibited acceptable test-retest reliability (Spearman ρ = 0.630; 95% CI, 0.43–0.77), and each domain-specific item exhibited poor to excellent reliability (ρ = 0.55–0.86), except lower reliability for transport and other sedentary behavior (ρ < 0.38; 95% CI, 0.13–0.58), as measured according to the Gardiner study criteria.^17^

Because the distribution of sedentary behavior was skewed, total sedentary time was calculated and then transformed into categorical variables with four levels (quartiles). The domains were dichotomized using median minutes per day spent viewing television (high [≥120 min] or low [<120 min]), using a computer (no [0 min] or yes [>0 min]), reading (no [0 min] or yes [>0 min]); socializing (≥30 min or <30 min), on transport (≥17.14 min or <17.14 min), and engaging in hobbies (no [0 min] or yes [>0 min]). The item of other sedentary time was combined with hobbies, because participants often reported other sedentary activities as hobbies.

#### Covariates

The covariates included age, gender, marital status (married or other), job status (employed or unemployed), education level (up to high school or college degree or more), residential area (metropolitan or nonmetropolitan), living status (alone or with family), and body mass index (BMI; self-reported and calculated using height and weight), which was dichotomized into non-overweight (<24 kg/m^2^) and overweight (≥24 kg/m^2^) according to Taiwanese cut-off points.^19^

We also included time spent in leisure time physical activity (LTPA) as a potential confounder. LTPA was assessed using the fourth part of the Taiwan version of the International Physical Activity Questionnaire long version (IPAQ-LV).^20^ The Taiwan version of the IPAQ-LV was reported to be a reliable instrument for assessing LTPA levels in Chinese-speaking older adults.^21^ The total times spent in vigorous-intensity leisure-time activity, moderate-intensity activity, and walking were calculated according to frequency (number of days in the last 7 days) and duration (minutes per day) and dichotomized into sufficient LTPA (≥150 min/week) and insufficient LTPA (<150 min/week), as defined by public health guidelines.^22^^,^^23^ The test-retest reliability of the Taiwan version of the IPAQ-LV was 0.80. The ICCs of the content validity indices were that the language equivalence ICC = 0.992 and that meaning similarity = 0.994 between the English and Chinese IPAQ-LS versions, respectively.^20^

### Statistical analyses

The data of 1046 older adults who provided complete information for the study variables were analyzed. A chi-square test was performed to identify proportional differences in sample characteristics between the type 2 diabetes categories. A Mann-Whitney U test was also performed to calculate significant differences between the mean (standard deviation [SD]) of total sedentary time and domain-specific sedentary behavior between the type 2 diabetes groups. Because the distribution of sedentary behavior was skewed, binary logistic regression was conducted to estimate the odds ratios (ORs) and 95% CIs of the associations between total and domain-specific sedentary behavior with type 2 diabetes. Analyses were conducted using SPSS Version 24.0 (IBM Corp, Armonk, NY, USA) with the level of significance set at *P* < 0.05.

## RESULTS

Table 1 contains the sociodemographic characteristics in the total sample and by status of type 2 diabetes among older adults. The mean (SD) age of the respondents was 73.6 (6.72) years. Overall, 46.9% of respondents were men, 75.7% were married, 80.8% were unemployed, 76.7% had an educational level of up to high school, 51.1% lived in a metropolitan area, 86.9% lived with family, 60.6% engaged in insufficient LTPA, and 41.9% were overweight. A total of 17.4% of respondents reported type 2 diabetes. Chi-square test analysis revealed proportional differences in education level (*P* = 0.04) and BMI status (*P* = 0.001).

**Table 1.** Characteristics of participants

Variable	Category	Total sample, *n* (%)		No type 2 diabetes, %	Presence of type 2 diabetes, %	*P* value
		
		*n* = 1046 (100)		*n* = 863 (82.6)	*n* = 183 (17.4)	
Age	Mean (SD)	73.6 (6.72)				
Gender	Men	491	(46.9)	80.4	19.6	0.10
	Women	555	(53.1)	84.3	15.7	
Marital status	Married	791	(75.7)	82.8	17.2	0.65
	Other	255	(24.4)	81.6	18.4	
Job status	Employment	201	(19.2)	83.1	16.9	0.06
	Not employment	845	(80.8)	82.4	17.6	
Education level	College degree or more	244	(23.7)	86.9	13.1	0.04
	Up to high school	802	(76.7)	81.2	18.8	
Residential area	Metropolitan	534	(51.1)	82.8	17.2	0.05
	Non-metropolitan	512	(48.9)	82.2	17.8	
Living status	Alone	137	(13.1)	82.5	17.5	0.99
	With family	909	(86.9)	82.5	17.5	
LTPA (min/week)	Sufficient (≥150)	449	(39.4)	83.9	16.1	0.17
	Insufficient (<150)	597	(60.6)	80.6	19.4	
BMI (kg/m^2^)	Non-overweight (<24)	608	(58.1)	85.7	14.3	<0.001
	Overweight (≥24)	438	(41.9)	78.1	21.9	

BMI, body mass index; LTPA, leisure time physical activity; SD, standard deviation.

Table 2 shows the means of sedentary behavior variables. The mean (SD) overall sedentary time was 283.03 (173.23) min/day. Among the six domains, the average time spent viewing television was 138.06 (112.2) min/day, time spent using a computer was 26.23 (65.02) min/day, time spent reading was 224.0 (372.74) min/day, time spent socializing was 49.65 (53.06) min/day, time spent on transport was 27.02 (34.2) min/day, and time spent engaging in hobbies was 9.10 (28.88) min/day. A Mann-Whitney U test was used to determine the differences in total and domain-specific sedentary time between the type 2 diabetes groups. A significant difference in type 2 diabetes status was observed in only the television viewing time domain (*P* = 0.002); no significant association with type 2 diabetes was found with total sedentary time (*P* = 0.13), computer use (*P* = 0.22), reading (*P* = 0.22), socializing (*P* = 0.78), transport (*P* = 0.16), or hobbies (*P* = 0.11).

**Table 2.** Domain-specific sedentary behaviors by type 2 diabetes

Variable (min/day)	Total sample Mean (SD) (*n* = 1046)	No type 2 diabetes Mean (SD) (*n* = 863)	Presence of type 2 diabetes Mean (SD) (*n* = 183)	*P* value
Total sedentary time	283.03 (173.23)	277.98 (168.66)	306.85 (192.05)	0.13
TV viewing	138.06 (112.2)	131.88 (105.63)	167.21 (135.66)	0.002
Computer use	26.23 (65.02)	26.48 (62.15)	25.05 (77.32)	0.22
Reading	224.0 (372.74)	225.72 (367.98)	215.90 (395.37)	0.22
Socializing	49.65 (53.06)	50.04 (53.94)	47.85 (48.76)	0.78
Transport	27.02 (34.2)	26.57 (34.37)	29.15 (33.41)	0.16
Hobbies	9.10 (28.88)	9.69 (30.12)	6.32 (21.97)	0.11

SD, standard deviation.

Table 3 shows the adjusted ORs and 95% CIs of type 2 diabetes according to sedentary behavior variables. The results indicated that no significant associations were found between total sedentary time and risk of type 2 diabetes after controlling for confounding factors. Regarding the associations between domain-specific sedentary behavior and risk of type 2 diabetes, the results indicated that viewing more television (≥120 min/day) was associated with higher odds of type 2 diabetes (OR 1.56; 95% CI, 1.10–2.21). However, no significant associations were observed between other domains of sedentary behavior and odds of type 2 diabetes.

**Table 3.** Odds ratios for type 2 diabetes by total and domain-specific of sedentary behaviors in older adults

Sedentary behavior variable	Total sample		OR (95% CI)	*P* value
Total sedentary time (min/day)	*n*	(%)		
Quartile 1 (0 to 152.13)	261	(25.0)	1.00 (Ref.)	
Quartile 2 (152.14 to 248.56)	258	(24.7)	1.28 (0.80–2.05)	0.31
Quartile 3 (248.57 to 389.99)	264	(25.2)	1.21 (0.74–1.98)	0.45
Quartile 4 (≥390.00)	263	(25.1)	1.41 (0.86–2.30)	0.18
TV viewing (min/day)				
Low (<120)	439	(41.1)	1.00 (Ref.)	
High (≥120)	616	(58.9)	1.56 (1.10–2.21)	0.01
Computer use (min/day)				
0	736	(70.4)	1.00 (Ref.)	0.43
>0	310	(29.6)	0.85 (0.56–1.29)	
Reading (min/day)				
0	544	(52.2)	1.00 (Ref.)	0.46
>0	502	(48.0)	0.87 (0.60–1.26)	
Socializing (min/day)				
Low (<30)	397	(38.0)	1.00 (Ref.)	0.46
High (≥30)	649	(62.0)	0.88 (0.62–1.24)	
Transport (min/day)				
Low (<17.14)	508	(48.6)	1.00 (Ref.)	0.17
High (≥17.14)	538	(51.4)	1.28 (0.90–1.83)	
Hobbies (min/day)				
0	881	(84.2)	1.00 (Ref.)	0.19
>0	165	(15.8)	0.72 (0.44–1.18)	

CI, confidence interval; OR, odds ratio; TV, television.

## DISCUSSION

This is one of the first studies to investigate the associations of total and domain-specific sedentary time with risk of type 2 diabetes in older adults in an Asian setting. The main finding of the present study is that only more time spent viewing television was found to be positively related to the risk of type 2 diabetes in Taiwanese older adults, with no significant associations observed in total sedentary time or time spent using a computer, reading, socializing, on transport, or engaging in hobbies. Therefore, these findings may have crucial implications for policymakers or intervention designers, suggesting that effective strategies to reduce television viewing time should be prioritized to prevent and manage the risk of type 2 diabetes in older adults.

The association demonstrated between more time spent viewing television and risk of type 2 diabetes in older adults, even after controlling for LTPA and other confounding factors, is consistent with the results of previous studies in adult populations.^9^^,^^10^^,^^12^ Specifically, our study revealed that watching television for more than 2 hours per day was positively associated with the risk of type 2 diabetes in older adults, which is also consistent with previous findings.^11^^,^^12^ Two hours or more of television per day has been reported as a critical cut-off point for health that contributes to increased risk of not only type 2 diabetes^11^^,^^12^ but also obesity, cardiovascular diseases, and all-cause mortality.^24^^,^^25^ Several possible explanations exist for this result. First, viewing television is a behavior characterized by few breaks and low energy expenditure,^26^^–^^28^ which can cause obesity, weight gain, and increased risk of type 2 diabetes.^29^ In particular, energy expenditure was found to decrease with age because of decreased basal metabolic rates in older adults.^30^ Therefore, prolonged television viewing could increase the risk of type 2 diabetes, particularly in older adults. Another possible reason is that television viewing is associated with other unhealthy behavioral risk factors, such as alcohol consumption and unhealthy eating (eg, snacks, processed meat, and fewer vegetables),^8^ which may increase the risk of type 2 diabetes. Thus, this finding may reveal the importance of restricting television viewing time to less than 2 hours per day not only for younger adults, but also for older adults, and may provide evidence for future sedentary behavior guidelines for disease prevention among older adults.

Another noteworthy finding was that total sedentary time was not associated with the risk of type 2 diabetes in older adults. A possible reason for this result is that domain-specific sedentary behavior time might be more critical than total sedentary time in influencing the risk of type 2 diabetes because different domains may contribute to different health outcomes.^31^^,^^32^ Thus, this result might guide the development of more effective domain-specific interventions for older adults. These results demonstrate the significance of examining domain-specific sedentary behavior^14^^,^^15^ and further suggest that future studies should investigate the associations of specific domains of sedentary behavior with other health outcomes.

Our results indicated that transport-related sedentary time was not related to the risk of type 2 diabetes in older adults, which contradicts a previous study of adults.^12^ A possible explanation for this result is that older adults may spend less time traveling (eg, commuting to work) and more time in neighborhood settings^33^ compared with the younger adult population. Another explanation could be that older adults in Taiwan, or at least those included in the sample of our data, may spend less time traveling (less than 30 min/day) than their counterparts in Western countries.^34^^,^^35^ The results of this study also indicated that time spent using a computer was not associated with type 2 diabetes. A possible reason for this could be the low prevalence of computer use among older adults.^36^ Other findings in this study indicate that the domain-specific sedentary behaviors of reading, socializing, and engaging in hobbies were not significantly associated with the risk of type 2 diabetes. A possible reason for these results is that the proportions of respondents who engaged in these behaviors were relatively low. Another possible explanation could be that energy expenditure for these activities is greater than that for watching television,^37^ so these other sedentary behaviors may not contribute to the risk of type 2 diabetes as much as viewing television does. An intervention study had demonstrated that interrupting sitting time with short bouts of light- to moderate-intensity walking led to significant reductions in postprandial glucose and insulin levels^38^; therefore, in an effort to reduce the risks of type 2 diabetes, this intervention strategy may contribute to health benefits in older adults.

Several limitations of the present study should be considered. First, the cross-sectional nature of these findings limits the conclusions that can be drawn from them because a causal link between sedentary behavior components and the risk of type 2 diabetes cannot be assumed. Second, the main measurements (namely, sedentary behavior, LTPA, and status of type 2 diabetes) were self-reported and could be subject to bias.^39^ Third, we did not measure other confounding variables, such as diet, alcohol consumption, and smoking, that might have confounded our results. Fourth, our study data were not a nationally representative sample because responses were limited to two localities and relied on a telephone-based survey. Moreover, including segments of the population that did not have a household telephone (approximately 5.3% in 2013)^40^ was impossible.

### Conclusion

These findings suggest that excessive television viewing (more than 2 h/day), a specific domain of sedentary behavior, may increase the risk of type 2 diabetes among older adults more than total sedentary behavior does.
